# Adding value to strawberry agro-industrial by-products through ultraviolet A-induced biofortification of antioxidant and anti-inflammatory phenolic compounds

**DOI:** 10.3389/fnut.2022.1080147

**Published:** 2022-12-07

**Authors:** Esteban Villamil-Galindo, Marilena Antunes-Ricardo, Andrea Marcela Piagentini, Daniel A. Jacobo-Velázquez

**Affiliations:** ^1^Instituto de Tecnología de Alimentos, Facultad de Ingeniería Química, Universidad Nacional del Litoral, Santa Fe, Argentina; ^2^Consejo Nacional de Investigaciones Científicas y Técnicas (CONICET), Santa Fe, Argentina; ^3^Tecnológico de Monterrey, The Institute for Obesity Research, Monterrey, Mexico; ^4^Tecnológico de Monterrey, Escuela de Ingeniería y Ciencias, Monterrey, Mexico; ^5^Tecnológico de Monterrey, The Institute for Obesity Research, Zapopan, Mexico; ^6^Tecnológico de Monterrey, Escuela de Ingeniería y Ciencias, Zapopan, Mexico

**Keywords:** biofortification, revalorization, UV radiation, circular economy, ellagitannins, postharvest abiotic stresses

## Abstract

**Background:**

The revalorization of agro-industrial by-products by applying ultraviolet A (UVA) radiation to biofortify with phenolic compounds has been studied in recent times, showing improvements in the individual and total phenolic content and their bioactivity. Therefore, the main aim of this work was to optimize the biofortification process of phenolic compounds by UVA radiation to strawberry agro-industrial by-products (RF). Moreover, the effect of UVA radiation on the potential biological activity of the phenolics accumulated in RF due to the treatment was also determined.

**Methods:**

The assays followed a factorial design with three variables at three levels: UVA dose (LOW, MEDIUM, and HIGH), storage temperature (5, 10, and 15°C), and storage time (0, 24, 48, and 72 h). At each experimental condition, phenylalanine ammonia-lyase (PAL) and polyphenol oxidase (PPO) enzymatic activities, total phenolic compound content (TPC), phenolics profile (TPC_HPLC_), and agrimoniin content (AGN) were evaluated; and the optimal UVA dose, storage time, and temperature were determined. *In vitro* bioaccessibility of the accumulated phenolic compound was studied on RF tissue treated with UVA at optimal process conditions. The digested extracts were tested for antiproliferative activity in colorectal cancer cells, cellular antioxidant capacity, and anti-inflammatory activity.

**Results:**

The results showed that applying UVA-HIGH (86.4 KJ/m^2^) treatment and storing the tissue for 46 h at 15°C increased PAL activity (260%), phenolic content (240%), and AGN (300%). The biofortification process improves the bioaccessibility of the main phenolic compound of RF by 9.8 to 25%. The digested optimum extract showed an IC50 for HT29 and Caco-2 cells of 2.73 and 5.43 μg/mL, respectively, and presented 60% cellular antioxidant capacity and 30% inhibition of NOX production.

**Conclusion:**

The RF treated with UVA is an excellent source of phenolic compounds; specifically, ellagitannins and the UVA radiation proved to be efficient in biofortify RF, significantly improving the phenolic compounds content and their bioactive properties with adequate bioaccessibility, adding value to the strawberry agro-industrial by-products.

## Introduction

Post-harvest processing of fruit and vegetables generates two types of agro-industrial by-products: avoidable and non-avoidable. Avoidable by-products are generated due to poor post-harvest handling during storage, processing, and transportation. On the other hand, non-avoidable by-products are derived from the conditioning of fruits and vegetables where the non-usable or non-edible parts of the product are eliminated (peels, seeds, leaves, stalks, or products in bad condition) (1). Agro-industrial by-products represent a significant challenge in their management and disposal in developed and developing countries (1). In the case of strawberry agro-industrial packaging, non-avoidable by-products can represent 7–20% of production (2). Different studies have been performed to describe the composition and the use of agro-industrial by-products in food and nutraceutical industries due to the composition rich in bioactive compounds such as vitamins, amino acids, dietary fiber, and secondary metabolites, in particular phenolic compounds (3, 4). The different strategies for the use of agro-industrial by-products allow the transformation from linear to circular processes, reducing the carbon footprint, maximizing the use of resources, offering environmental and economic benefits, and improving efficiency in developing countries’ agro-industries (4).

The polyphenolic distribution in strawberries depends on the crop’s tissue, variety, and environmental factors. Nevertheless, the major phenolic compounds in the strawberry plant have been reported as ellagitannins, phenolic acids, and flavonoids such as anthocyanins and quercetin glycosides (5). These metabolites offer different physiological functions to plants and are synthesized in response to different exogenous stimuli, one of the most common is UV radiation (6). The high energy rate of ultraviolet radiation can generate damage at the cellular level. However, the response of plants is generated depending on the dose and type of radiation they receive. The electromagnetic spectrum of UV radiation is between 100 and 400 nm, usually subdivided into 3 regions UVA (315–400 nm), UVB (280–315 nm), and UVC (100–280) (7), having different elicitor effects on plants. It has been reported that UV radiation activates enzymes related to the biosynthesis and utilization of phenolic compounds and depending on the biosynthesis rate and utilization rate, accumulation of phenolics can be observed in the tissue (8).

Obtaining health-promoting compounds from agro-industrial by-products is one strategy used to revalue agro-industrial by-products (2). Likewise, agro-industrial by-products can also be used as biofactories of phenolic compounds by applying different abiotic stresses that modulate their secondary metabolism, obtaining significant increases in the concentration of phenolic compounds (9). UV radiation may trigger phenylpropanoid metabolism in plant tissues. For instance, increases of up to 500% in total phenolic content have been reported in carrots (8), and a 700% increase in quercetin in red prickly pears (10). However, there are no specific UV radiation conditions to induce the accumulation of phenolics since each plant tissue responds differently to abiotic stresses.

To incorporate phenolic compounds extracted from agro-industrial by-products in different food or dietary supplement formulations, it is necessary to study their behavior in human metabolism. The cytotoxicity of phenolic compounds has been studied in healthy cell lines with different metabolic functions (11) and in cell cultures of different types of cancer and their antioxidant and anti-inflammatory activity (12). Various bioactivities of phenolic compounds obtained from strawberries and other plant parts have been reported in experimental *in vitro* studies, showing their potential to be a source of nutraceutical compounds (13). However, there is no report on the bioactivity of these compounds after the digestion process, as their content and stability are significantly affected by the type of matrix in which they are found and the gastrointestinal process. There is no report in the literature regarding the application of UV radiation to improve phenolic content from strawberry agro-industrial by-products and evaluate their bioaccessibility and bioactivity. Therefore, the main objective of this work was to add value to strawberry by-products by determining the optimal conditions of UVA radiation, storage temperature, and time to increase phenolic compounds’ content and assess their stability during digestion, cytotoxicity, and potential biological activity.

## Materials and methods

### Plant material and reagents

The by-products of strawberry (RF) (*Fragaria* × *ananassa* Duch) were obtained during postharvest industrial conditioning of strawberries from a field at Coronda, Argentina (31°58′00″S 60°55′00″W). The by-products of RF consist of sepals, peduncles, and fruit remains. The RF was packed in 40 μm polypropylene bags and stored at –80°C. Before assays, the samples were freeze-dried (5.2 % moisture content) and ground to a particle size <1 mm.

Polyvinylpolypyrrolidone (PVPP), Folin-Ciocalteu reagent, 2′,7′-dichlorodihydrofluorescein diacetate (DCFH-DA), pepsin from porcine gastric mucose (E.C.3.4.23.1 800–2500 units/mg protein), pancreatin from porcine pancreas (8XUSP), fluorescein, 2,2′-azobis(2-amidinopropane) dihydrochloride (AAPH), dichlorofluorescein diacetate and lipopolysaccharides (LPS), acetonitrile (HPLC grade), methanol (HPLC grade), ferulic acid, gallic acid, ellagic acid, procyanidin, quercetin, kaempferol, and albumin standards were obtained from Sigma Chemical Co. (St. Louis, MO, USA). DL-dithiothreitol (DTT) was purchased from Merck KGaA (Darmstadt, Germany). Sodium acetate trihydrate and potassium phosphate dibasic were acquired from Cicarelli Reagents S.A. (Santa Fe, Argentina). Fetal bovine serum and antibiotics for cell culture were from GIBCO (Grand Island, NY). Celltiter 96 aqueous one solution cell proliferation assay and Griess reagent were obtained from Promega (Madison, WI).

### Ultraviolet a radiation and storage conditions

Ultraviolet A (UVA) radiation treatment was carried out in a climate chamber (Memmert, Germany) equipped with two 8 W UVA lamps with a 320–400 nm spectrum range. The irradiation doses tested were 28.8 KJ/m^2^ (UVA-LOW), 57.6 KJ/m^2^ (UVA-MEDIUM), and 86.4 KJ/m^2^ (UVA-HIGH), determined with a UVA dosimeter (model 501, Solar Light Co., PA, USA). The sample was inverted in the middle of the process to ensure a homogeneous UVA radiation dose. The RF was exposed to UVA radiation for 1, 2, and 3 h at 20°C. After each treatment, the UVA-treated RF (RF-E) samples were packed in polypropylene boxes covered with PVC film (20 g). The UVA-treated samples (RF-E) and the control samples (RF-N, untreated RF tissue), equally packed, were stored at 5, 10, and 15°C, 90% RH, for 24, 48, and 72 h, to compare the effect of temperature storage stress.

### Enzymatic activity determinations

#### Phenylalanine ammonia-lyase activity

The phenylalanine ammonia-lyase (PAL) activity was measured according to Van de Velde et al. (14) with some modifications. Five grams (5 g) of strawberry by-product with 10 mL of extractant solution (phosphate buffer 100 mmol L^–1^ pH 8, EDTA 2 mmol L^–1^, PVPP 30 g L^–1^, DTT 7 mmol L^–1^, Triton X-100 0.1% v/v) were homogenized for 30 s and stirred for 45 min at 4°C. The mixture was centrifuged at 12,000 × *g* for 20 min at 4°C, and the supernatant was used to assess PAL activity. The reaction mixture was 1060 μL of Tris–HCl 100 mmol L^–1^ pH = 8.8, 530 μL phenylalanine 50 mmol L^–1^, and 150 μL of enzymatic extract. The reaction mixture was incubated at 37°C for 1 h, the reaction was stopped with 260 μL of TCA 10 g L^–1^, and centrifuged at 12,000×*g* for 10 min. Cinnamic acid production was measured by the absorbance change at 290 nm (Genesis 10S UV–Vis spectrophotometer, Thermo Scientific, Germany). The results were expressed as the change of absorbance by an hour and milligram of protein (ΔA mg protein^–1^h^–1^).

#### Polyphenol oxidase activity

The technique adapted from Rodríguez-Arzuaga et al. (15) was used to determine the polyphenol oxidase (PPO) activity. Strawberry by-product (5 g) were homogenized and stirred (1 h at 4°C) with 10 mL of extraction solution (phosphate buffer 100 mmol L^–1^ pH = 6, PVPP 30 g L^–1^, Triton X-100 0.1% v/v, NaCl 1 mol L^–1^). The mixture was centrifuged at 12,000 × *g* for 15 min at 4°C, and the supernatant was used to assess PPO activity. The reaction mixture (900 μL of distilled water, 200 μL of pyrocatechol 200 mmol L^–1^, 150 μL of phosphate buffer 1 mol L^–1^ pH = 6, and 250 μL of extract) was incubated for 1 h at 37°C. Absorbance variation was measured at 410 nm, and the results were expressed as (ΔA mg protein^–1^h^–1^).

#### Protein determination in enzyme extracts

The Lowry et al. (16) method was applied to determine the protein content in the PAL and PPO extracts. Enzymatic extract (200 μL) in adequate dilution was added to 2 mL of reagent C (100 parts of 3.0% Na_2_CO_3_, 0.4% NaOH, 4% sodium tartrate, and 1 part of reagent B: 2% CuSO_4_⋅5 H2O), and incubated at room temperature for 10 min. Then, 200 μL of Folin-Ciocalteu reagent was added (1 N), the mixture was vigorously shaken, incubated at 20°C for 30 min, and the absorbance was determined at 660 nm. A calibration curve was generated using 1 mg L^–1^ of albumin. The results were expressed as mg of protein per g of sample (mg g^–1^).

### Phenolic compound characterization

#### Phenolic compounds extraction

Phenolic compounds from RF were extracted as previously described by Villamil-Galindo et al. (17). Briefly, 1 g of RF was sonicated for 15 min (T40, Teslab, Buenos Aires, Argentina) with 5 mL of 80% methanol with 0.5% formic acid and centrifuged at 12000×*g* for 20 min (Neofuge 18R Heal Force refrigerated centrifuge, Shanghai, China). The supernatant was collected, and the pellet was reextracted under the same conditions. The supernatants were mixed and brought up to 10 mL. The extracts were stored at –20°C until analyses.

#### Determination of phenolic compound profiles

The phenolic compounds profile was determined in each RF sample using an LC-20AT high-performance liquid chromatograph with a diode array detector and Lab Solutions for data processing and control software (Shimadzu Co., Kyoto, Japan). Separation was performed on a Gemini 5 μ C18 110 Å 250 × 4.6 mm hybrid reverse phase column attached to a guard column (Phenomenex Inc, CA, USA). Determinations were performed according to Villamil-Galindo et al. (2).

Identification of phenolic compounds was performed by retention times and UV-Vis absorption spectra compared with commercial phenolic compounds standards. The integrated areas of the obtained peaks (mAU * min) of the standard phenolic compounds were plotted as a function of their concentrations. The equations for calculating the extracts’ content were obtained from each linear regression. The results were expressed as g of phenolic compound kg^–1^ by-product.

#### Total phenolic content

The total phenolic content (TPC) determination of the RF samples was performed according to Villamil-Galindo et al. (2) with some modifications using the Folin-Ciocalteu method. Twenty microliters (20 uL) of the extracts, 100 uL Folin-Ciocalteu reagent (0.66 N), 100 uL of sodium carbonate solution (10%), and 780 uL of distilled water were mixed and allowed to react at 20°C for 30 min in triplicate. Then, the mixture was put into a 96-well plate, and the absorbance was measured at 765 nm in the microplate reader (Synergy HT, Bio-Tek, Winooski, VT, USA). The results were expressed as g of gallic acid equivalents (GAE) per kilogram of strawberry by-product (g GAE kg^–1^).

### *In vitro* gastrointestinal digestion assay

The assay was performed according to the methodology proposed by Flores et al. (18). It consists of three distinct stages: oral phase (Simulated Saliva Fluid, SF), gastric phase (Simulated Gastric Fluid, GF), and intestinal phase (Simulated Duodenal Fluid, DF, and bile solution, BF) (IF). The composition of the different phases was prepared according to Supplementary Table 1.

Samples (RF-N and RF-E) were weighted into a stoppered 125 mL Erlenmeyer flask at a ratio of 1:3 w/v with SF. The SF fluid containing human a-amylase (Sigma-Aldrich Inc., St. Louis, MO, USA) was added, and the sample was incubated at pH 6.8 ± 0.2 for 2 min. Then, 6 mL of GF containing porcine gastric pepsin Sigma-Aldrich Inc. (St. Louis, MO, USA) was added, pH adjusted to 1.3 ± 0.2, and the sample was re-incubated for 1 h. The final step in the digestion procedure included the addition of 6 mL DF and 3 mL BF containing porcine pancreatin (Sigma-Aldrich Inc., St. Louis, MO, USA). The pH was adjusted to 7.0 ± 0.2, and samples were incubated for another 1 h at 37°C in a shaker (New Brunswick Scientific, Eppendorf). Aliquots (0.5 mL) were collected after each phase, and the samples were immediately placed into a water bath at 90°C for 3 min to inactive the enzymes. Then, they were centrifuged at 4°C for 15 min at 9000 × *g* (Eppendorf Centrifuge 5804R, Germany), and the supernatant was collected. Each digestion was performed in triplicate. Each phase obtained was analyzed by HPLC-DAD, and the final phase (intestinal phase) was used to perform the cell culture assays. The results were expressed as a percentage (%) of bioaccessibility according to the ratio between the phenolic compound concentration in the intestinal fraction and their initial concentration in the raw material or dried extract (Equation 1).


(1) $$\mathrm{Bioacessibility}(\%)=\frac{\mathrm{Phenolic compounds intestinal phase}}{\mathrm{Phenolic compound in raw material}\,(\mathrm{Dried extract})}\times 100$$

### Cell culture

Cell lines, human colorectal adenocarcinoma cells (Caco-2 and HT29), mouse macrophage (RAW 264.7), and adult primary dermal fibroblast; (HDFa) were purchased from the American Type Culture Collection (Manassas, VA, USA). The cells were maintained in modified eagle medium (DMEM) supplemented with 5% fetal bovine serum (FBS) and 1% antibiotic, streptomycin (10,000 μg/mL), at 37°C and under 5% CO_2_ in a humidified incubator.

### Anti-inflammatory potential

The potential anti-inflammatory activity of the resuspended digestion of freeze-dried raw material was tested at a previously determined concentration of 1.75 μg/mL of digested RF. RAW 264.7 cell line was used to measure nitric oxide (NO) production induced by Lipopolysaccharides (LPS) from *Salmonella enterica* serotype typhimurium (L7261, Sigma-Aldrich, St Louis, MO; USA). This macrophage cell line is a cell type that responds quickly to stimuli, thus facilitating the screening of the immune response to the presence of phenolic compounds present in RF digests, which is vital as it is a source of bioactives that is not usually edible. The assay was performed according to Moreno-García et al. (19)., with some modifications. Prior, 100 μL with 5 × 10^5^ cells were seeded in each well of a 96-well cell culture plate. After 24 h, 50 μL of each sample was placed in triplicate and incubated for 4 h at 37°C, followed by the addition of 50 μL LPS (10 μg/mL) to half of the wells to stimulate the inflammatory process. Half of the wells were used as a control with 50 μL DMEM medium and incubated for 18 h at 37°C.

The NO production was determined by Griess Reagent System (Promega, Madison, WI, USA) following the manufacturer’s instructions. Briefly, 100 μL of each sample and the controls were taken and placed in a new 96-well plate, and 10 μL of component A (10 mg/mL) (Sulfanilic acid) was added to each well, shaking the plates for 10 min, followed by the addition of 10 μL of component B(1 mg/mL) [N-(1-naphthyl) ethylenediamine dihydrochloride]. The quantification was done using a nitrites standard curve (0.78–50 μM, R^2^ 0.99) measuring the absorbance at 550 nm, considering cells without LPS stimuli and cell viability. The initial plate was used to determine the RAW 264.7 viability using CellTiter 96 Aqueous One Solution Cell Proliferation Assay (Promega, Madison, WI, USA). The absorbance was measured at 490 nm to determine cell viability. The data were expressed as a percentage of inhibition compared to the positive control plate and cell viability.

### Antioxidant cellular activity

During the digestive process of RF as a source of phenolic compounds, their residence time in the colon is long, so it is important to study the anti-radical capacity of these phenolic compounds in colon cells. To measure the level of reactive oxygen species (ROS) produced by the cells in the presence of different digested extracts (from RF-N and RF-E samples) at 2.2 μg/mL, Caco-2 cells suspension was seeded at a density of 5 × 10^5^ cells/mL on a black 96-well microplate in 100 μL growth medium per plate 24 h before the assay. To determine the antioxidant cellular activity of different extracts, the methodology proposed by Moreno-García et al. (19) was used with some modifications. After 24 h, the medium was removed, and each well was carefully washed with PBS pH 7.4 (1×). Samples were diluted in DMEM medium to 2× concentration, then 2′,7′-dichlorodihydrofluorescein diacetate (DCFH-DA, 120 μM) fluorescent agent was added in a final concentration of 60 μM. The mixture was added at a volume of 100 uL in each well. For negative and positive control, 100 μL of DMEM medium with 60 μM DCFH was placed. For the reaction blank, 100 μL of DMEM was added. The plate was incubated at 37°C for 20 min. Subsequently, the reaction solutions were removed from each well, and the cells were washed twice with PBS. To induce the pro-oxidant stimuli in the cells, 100 μL/well of APPH [2,2′-Azobis(2-methylpropionamidine) dihydrochloride] at 500 μM was added, except for blank (100 μL of DMEM) and negative control wells (100 μL of PBS). Fluorescence emitted at 538 nm with excitation at 485 nm was measured every 2 min for 120 min at 37°C. The results were expressed following (Equation 2)


(2) %(CAA)=1−(∫SA/∫CA)

where, ∫*SA* is the integrated area under sample fluorescence versus time curve and, ∫CA is the integrated area from the control curve.

### Antiproliferative activity

The digested fraction of RF-N and RF-E having a long residence time in the colon, its antiproliferative activity was evaluated in colon cancer cells (Caco-2 and HT29), and healthy control (HDFa) cells were used. The ratio of live and dead cells was determined by the methodology proposed by Pacheco-Ordaz et al. (20). Different extract concentrations were tested (0.0094-122 mg/mL). Caco-2, HT29, and HDFa were seeded in a 96-well plate in a suspension of 100 μL at 5 × 10^5^ cells/mL. After 24 h of incubation at 37°C, 100 μL of each extract was placed at the final concentrations, and the plates were incubated for 48 h at 37°C. Subsequently, 10 μL of CellTiter 96 Aqueous One Solution Cell Proliferation Assay (Promega, Madison, WI) was added in each well, and then, absorbance was measured at 490 nm in a microplate reader (Synergy HT, Bio-Tek, Winooski, VT, USA). The IC_50_ values (half maximal inhibitory concentration) for each sample were determined from the percentage viability versus concentration data; these were fitted to an asymmetric sigmoidal equation, and the appropriate concentration to inhibit 50% viability in the cells studied was calculated.

### Statistical analysis

The effect of UVA dose (UVA), storage time (ST), and temperature (TM) on PPO and PAL activities, TPC, TPC_HPLC_, and agrimoniin content (AGN) were determined by analysis of variance (ANOVA). Significant differences among treatment means were determined by Tukey’s test (*p* < 0.05). The response surface methodology (RSM) allowed modeling and optimizing (simultaneous optimization of several response variables) the biofortification process in phenolic compounds, using a second-order polynomial equation to model and obtain the coefficients of each response as a function of UVA, ST, and TM. The linear stepwise regression procedure was used to reduce the non-significant terms in the model, which was determined by the R^2^ and the lack of fit. Analyses were performed with STATGRAPHICS Centurion XV (StatPoint Technologies Inc., Warrenton. VA, USA).

## Results and discussion

### Effect of ultraviolet a dose, storage time, and temperature on the strawberry by-product biofortification process

The RSM allowed studying and modeling the effect of the UVA radiation dose, storage temperature (ST), and storage time (TM) on the enzymatic activity of PAL, PPO, total phenolic (TPC and TPC_HPLC_), and the agrimoniin content (AGN) of strawberry agro-industrial by-products (RF) (Supplementary Table 2 and Supplementary Equations 2–5).

According to the coefficients of determination (R^2^ 0.62–0.89) and the lack of fit test (p-value 0.10–0.52), the quadratic model best described the behavior of the different response variables studied (Supplementary Table 2).

#### Phenylalanine ammonia-lyase activity

**FIGURE 1:**
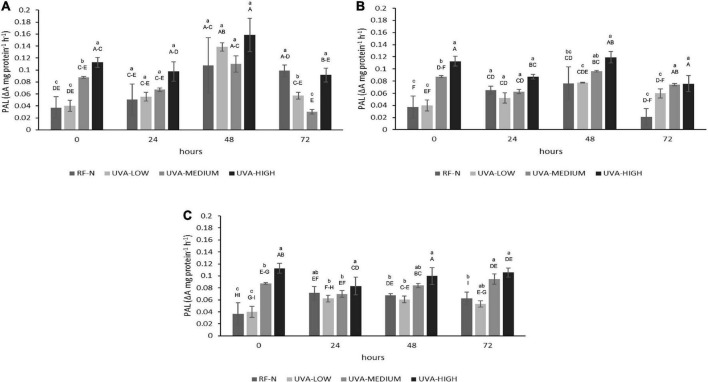
Phenylalanine ammonia-lyase (PAL) activity of UVA-treated strawberry by-products during storage at **(A)** 5°C, **(B)** 10°C, and **(C)** 15°C. RF-N: Strawberry agro-industrial by-product control. UVA-LOW: RF irradiated with 28.8 KJ/m^2^. UVA-MEDIUM: RF irradiated with 57.6 KJ/m^2^. UVA-HIGH: RF irradiated with 86.4 KJ/m^2^ Different lower-case letters mean significant differences (*p* ≤ 0.05) among treatments at the same storage time. Different capital letters mean significant differences (*p* ≤ 0.05) among different storage times.

Phenylalanine ammonia-lyase is a ubiquitous enzyme in plants; it catalyzes the first step in phenylpropanoid metabolism, accumulating antioxidant phenolic compounds. Using elicitors such as UVA radiation to increase ROS production in strawberry agro-industrial by-products plays a major role in activating the PAL enzyme (21). PAL activity was mainly affected by UVA (*p* < 0.001), ST (*p* < 0.01), and TM (*p* < 0.001), and the quadratic factors UVA^2^ (*p* < 0.05) and TM^2^ (*p* < 0.01) (Supplementary Table 2). Initially, the RF tissue showed an average PAL activity of 0.04 ΔA mg protein^–1^h^–1^ (Figure 1), which is 57% higher than reported for the whole strawberry fruit with 0.017 ΔA mg protein^–1^h^–1^ (14).

Immediately after UVA radiation treatments, PAL activity presented the highest increase (304%) for UVA-HIGH treatment, followed by UVA-MEDIUM (236%). Otherwise, there were no differences (*p* > 0.05) in the PAL initial activity with UVA-LOW. The physiological response of plants to UVA radiation varies significantly, and the mechanisms of response are not completely clear. What is known is that the absorption of different doses of UVA is mediated through photoreceptors present in different parts of the strawberry plant, cytochrome receptors (CRY), and phototropin receptors (PHOT) (22, 23). When the UVA photoreceptors are saturated, ROS are generated as the first signal, inducing the transcription of regulatory genes, leading to the expression of enzymes from the phenylpropanoid pathway such as PAL, triggering an increase in mitochondrial respiration, accelerating the metabolism of the plant tissue (22). These physiological events are likely occurring in the strawberry agro-industrial residue, which initially responds according to the intensity of the UVA dose.

During the different storage times and temperatures, the PAL activity varied significantly (Figure 1). There was a slight decrease in PAL for UVA-MEDIUM and UVA-HIGH samples after 24 h at 5°C (*p* < 0.05). However, after 48 h, the highest increases in PAL were determined at 5°C, for the control (RF-N, 290%), UVA-MEDIUM (297%), UVA-LOW (373%), and UVA-HIGH (427%), compared to the initial PAL activity of the RF control sample (RF-N, ST = 0). These results agreed with those previously reported for lettuce treated with UVA intensity of 19 μmol m^–2^ s^–1^, where storage at 16°C induced a 300% increase in PAL activity (24). RF samples stored at 10°C showed less PAL activity than those at 5°C. However, the highest PAL increases were obtained between 24 to 48 h of storage, with the RF-N and UVA-LOW samples showing similar (*p* > 0.05) increases of 200% and 117%, respectively, followed by UVA-MEDIUM (260%). Nevertheless, the highest increase was 322% with 0.12 ΔA mg protein^–1^h^–1^ at 10°C with UVA-HIGH. The RF-E with UVA-HIGH at 15°C showed PAL increments between 192 and 285% at 72 h of storage (0.08–0.11 ΔA mg protein^–1^h^–1^). These results disagree with the expected increase of enzyme activity with temperature. The optimal temperature for PAL activity is 37°C, and its temperature-dependence enzymatic activity has already been reported (25). However, the PAL enzyme is encoded by a multigene family that generates several isoforms of the enzyme, which may result in variability in each plant tissue. These results agree with those reported by Formica-Oliveira et al. (26) where the most significant PAL increases in stressed tissues are generated after 24–48 h of storage, followed by the accumulation of phenolic compounds in 48–72 h with a moderate rise in PAL activity.

#### Polyphenol oxidase

**FIGURE 2:**
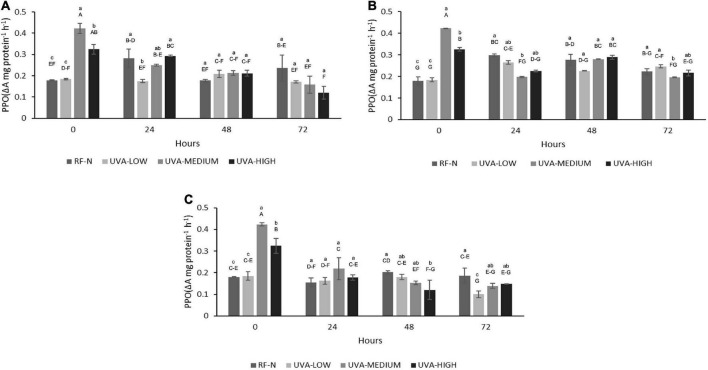
Polyphenol Oxidase (PPO) activity of UVA-treated strawberry by-products during storage at **(A)** 5°C, **(B)** 10°C, **(C)** 15°C. RF-N: Strawberry agro-industrial by-product control. UVA-LOW: RF irradiated with 28.8 KJ/m^2^. UVA-MEDIUM: RF irradiated with 57.6 KJ/m^2^. UVA-HIGH: RF irradiated with 86.4 KJ/m^2^. Different lower-case letters mean significant differences (*p* ≤ 0.05) among treatments at the same storage time. Different capital letters mean significant differences (*p* ≤ 0.05) among different storage times.

Ultraviolet A (UVA) (*p* < 0.01), ST (*p* < 0.05), and TM (*p* < 0.001) significantly affected PPO activity. Additionally, the interaction of the factors UVAxTM was significant (*p* < 0.001) on PPO (Supplementary Table 2), meaning the effect of UVA dose depends on TM value. The influence of different doses of UVA radiation on PPO is shown in Figure 2. UVA-LOW treatment did not exert significant changes in PPO activity compared with the control (0.18 ΔA mg protein^–1^h^–1^). Initially, UVA-MEDIUM treatment increased PPO activity by 236%, whereas the UVA-HIGH sample showed a PPO increase of 181%. The lower increase in PPO activity observed in UVA-HIGH samples could be explained in terms of a potential denaturation of the protein since excess energy from longer radiation times could generate a partial denaturation of the enzyme.

Moreover, after 24 h of storage (5°C), the PPO activity of RF-N increased by 57%, which remained relatively constant until 72 h. UVA-LOW samples maintained their PPO activity constant for up to 72 h. For RF-E UVA-MEDIUM and HIGH after 72 h, a decrease in PPO was observed, reaching up to 33% lower than the PPO of the initial RF-N. At 10°C and 24 h, RF-N and UVA-LOW showed increases in PPO activity between 66 and 48%, respectively. In contrast, the trend was toward a decrease in PPO compared to the initial treatment time for UVA-MEDIUM and UV-HIGH at 10°C and 24 h (Figure 2B). For the samples stored at 15°C, a reduction in PPO activity was determined for all treatments, especially in the UVA-HIGH treatment, which had an initial activity of 0.32 ΔA mg protein^–1^h^–1^, and it dropped to 0.12 ΔA mg protein^–1^h^–1^ after 48 h of storage. This behavior can be explained by the fact that the strawberry PPO enzyme has an average optimum pH and temperature of 6.2 and 35°C (27). In the case of strawberries, the average pH is between 3.4 and 3.5, which may have a synergistic effect with the different storage temperatures and radiation doses, controlling PPO activity by reducing the oxidation of *de novo* phenolic compounds synthesized (14). Similarly, Teoh et al. (28) showed a synergistic effect of low pH with high doses of UVC radiation in controlling the PPO activity of potato slices. Otherwise, Ding and Ling (29) reports that UVC doses of 0.04 KJ/m^2^ applied on banana peel induced enzymatic browning. On the other hand, plant tissues maintain a balance between *de novo* synthesis and utilization of phenolic compounds, which correlates with an increase in PPO activity (30). PPO had very slight increases for RF-E even after 72 h at 15°C for all UVA doses; the PPO activity of RF-E samples was lower than RF control samples (RF-N). However, Chen et al. (31) have already reported this behavior in plant tissues, where the PPO activity of irradiated lettuce at low doses (10 μmol m^–2^ s^–1^) decreased by almost 40%.

#### Total phenolic content

**FIGURE 3:**
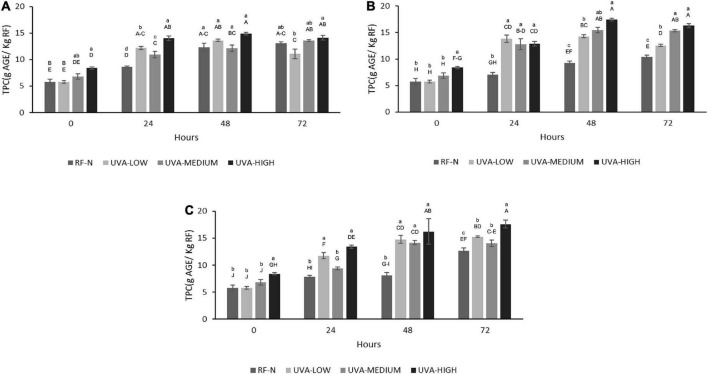
Total phenolic content (TPC) of UVA-treated strawberry by-products during storage at **(A)** 5°C, **(B)** 10°C, **(C)** 15°C. RF-N: Strawberry agro-industrial by-product control. UVA-LOW: RF irradiated with 28.8 KJ/m^2^. UVA-MEDIUM: RF irradiated with 57.6 KJ/m^2^. UVA-HIGH: RF irradiated with 86.4 KJ/m^2^. Different lower-case letter, means significant differences (*p* ≤ 0.05) between the different treatments at the same storage time. Different capital letter means significant differences (*p* ≤ 0.05) between different storage times.

The effect of UV radiation on different plants vary widely, depending on factors such as the stage of maturity of the plant or fruit, cultivar, type of crop, availability of carbon source in the plant (sugars and amino acids), and type and dose of radiation (17). For RF, the TPC was significantly affected (*p* < 0.01) by TM and TM^2^. The interaction term UVAxTM^2^ also affected TPC (*p* < 0.05) (Supplementary Table 2). The initial content of total soluble phenolic compounds of RF-N was 5.8 mg AGE/g RF. At ST = 0, the UVA-LOW treatment did not change TPC (Figure 3). However, the elicitor effect can be evidenced with the UVA-MEDIUM treatment with TPC 6.8 mg AGE/g. Likewise, following a dose-dependent behavior with UVA-HIGH, an increase of 46% in TPC (8.4 mg AGE/g) was achieved, correlating with that shown in the PAL (Figure 1), indicating that UVA stress generates the modulation of secondary metabolism in RF tissue through the synthesis of new phenolic compounds.

According to the TPC obtained at 5°C, the metabolism of phenylpropanoids remained active in the biosynthesis of phenolic compounds, with the most significant increases being observed after 48 h, achieving up to 158% more phenolic compounds for RF with UVA-HIGH (14.9 mg AGE/g) as compared to the initial control value. However, the most significant increase was determined after storing the UVA-HIGH samples at 10°C for 48 h, obtaining a TPC of 17.4 mg AGE/g, 142% higher than the control (9.3 mg AGE/g) after 48 h of storage, positively correlating with the 322% increase in PAL for the same period. A similar increase was achieved with UVA-HIGH treatment after 72 h at 15°C (17.6 mg AGE/G), showing that the increase in TPC was not affected by the storage temperature (*p* > 0.05). UVA is the major component of the solar UV spectrum, 95% UVA (315–380nm) and UVB (280–315 nm), despite being less harmful than UVB and UVC radiation. UVA radiation can penetrate plant tissue, generating physiological responses to control the stress condition, as observed in RF tissue at different doses and subsequent storage (32).

#### Phenolic compounds profile

The use of UVA radiation as an elicitor has been little studied. The phenolic biofortification with UVA radiation has been reported in a few plant tissues such as *Rosa hybrida* and *Fuchsia hybrida* (33), *Betula pendula* (32), *Daucus carota* (8), *Brassica oleracea* (34), *Vaccinium sect. Cyanococcus* cv “*Duke”* (35) and *Glycine max* sprout (36).

It has been reported that UVA radiation can affect the photosynthesis process in plant tissues and modulate the synthesis of specific phenolic compounds (37), and regarding by-products, Formica-Oliveira et al. (38) studied the effect of UVB and UVC radiation on broccoli by-products; and Sánchez-Rangel et al. (39) studied the effect of UVC on the biosynthesis of phenolics in carrot bagasse. The profile of phenolic compounds obtained by HPLC-DAD allowed the quantification and identification of 8 individual phenolic compounds (Tables 1A,B), with hydrolyzable tannins such as tetragalloyl glucose isomer (TGI) and agrimoniin (AGN) as the main compounds, ellagic acid derivatives such as ellagic acid pentoside (EAP) and free ellagic acid (EA) and flavonoids such as procyanidin tetramer (PACT), quercetin 3-O-glucuronide (Q3G) and Kaempferol 3-o-glucuronide (K3G). The effects of UVA, ST, and TM on the individual phenolic compounds are shown in Tables 1A,B. Moreover, the total sum of the identified phenolic compounds (TPC_HPLC_) was fitted to a second-order model with UVA (*p* < 0.001), ST (*p* < 0.001), and TM (*p* < 0.01) as the independent variables (Supplementary Equation 4). The TPC_HPLC_ variation was affected by the 3 studied factors, UVA, ST, and TM, and the interaction of the factors STxTM (<0.01) (Supplementary Table 2). The major compound, AGN, is of great interest because several authors have reported the multiple bioactivities with applications in the nutraceutical industry (40, 41). For this reason, it was also modeled and included in the multiple response optimization (Supplementary Equation 5). According to the results obtained, the UVA and TM affected (*p* < 0.01) the AGN content.

**TABLE 1A:** Changes in the individual phenolic compound concentration of strawberry by-products as affected by UVA dose, storage time, and temperature.

UVA	Storage temp (°C)	Storage time (h)	TGI (g/Kg)	EAP (g/Kg)	AGN (g/Kg)	EA (g/Kg)
RF-N	5	0	0.26 ± 0.04a	0.21 ± 0.01m-p	0.60 ± 0.01q	0.05 ± 0.003m
		24	0.14 ± 0.01d–g	0.20 ± 0.001n–p	0.90 ± 0.19n–p	0.09 ± 0.03 e-l
		48	0.13 ± 0.02e–h	0.24 ± 0.01i–n	0.93 ± 0.25 n–p	0.07 ± 0.01i–m
		72	0.13 ± 0.001e–i	0.25 ± 0.05i–n	0.77 ± 0.18pq	0.07 ± 0.003i–m
	10	0	0.26 ± 0.01a	0.21 ± 0.01m–p	0.60 ± 0.01q	0.05 ± 0.003 m
		24	0.10 ± 0.01h–k	0.23 ± 0.03k–p	1.25 ± 0.26 i–l	0.09 ± 0.02 e–j
		48	0.11 ± 0.01f–j	0.21 ± 0.01m–p	1.08 ± 0.02l–n	0.11 ± 0.003 b–f
		72	0.11 ± 0.02f–j	0.14 ± 0.02 q	0.79 ± 0.03pq	0.06 ± 0.002k–m
	15	0	0.26 ± 0.04a	0.21 ± 0.04m–p	0.60 ± 0.01q	0.05 ± 0.003m
		24	0.13 ± 0.001e–i	0.82 ± 0.01j–o	0.82 ± 0.19p	0.07 ± 0.01i–m
		48	0.14 ± 0.001d–f	0.21 ± 0.01m–p	1.33 ± 0.18 g–k	0.06 ± 0.01j–m
		72	0.13 ± 0.02d–h	0.23 ± 0.06 k–p	1.32 ± 0.07g–k	0.07 ± 0.04i–m
Low	5	0	0.15 ± 0.01c–e	0.23 ± 0.02k–p	0.84 ± 0.05op	0.08 ± 0.02f–l
		24	0.15 ± 0.02c–e	0.30 ± 0.01c–i	1.25 ± 0.03i–m	0.10 ± 0.03 b–h
		48	0.16 ± 0.02c–e	0.31 ± 0.04c–h	1.62 ± 0.05c–f	0.11 ± 0.01b–e
		72	0.09 ± 0.02jk	0.24 ± 0.02k–p	1.04 ± 0.07m–o	0.06 ± 0,02lm
	10	0	0.15 ± 0.01c–e	0.23 ± 0.02k–p	0.84 ± 0.05op	0.08 ± 0.02f–l
		24	0.08 ± 0.002k	0.23 ± 0.04k–p	1.08 ± 0.06l–n	0.07 ± 0.01h–m
		48	0.10 ± 0.001i–k	0.24 ± 0.01i–n	1.39 ± 0.01f–k	0.08 ± 0.01f–l
		72	0.11 ± 0.003f–j	0.31 ± 0.01c–g	1.51 ± 0.08c–h	0.10 ± 0.01b–g
	15	0	0.15 ± 0.01c–e	0.23 ± 0.02k–p	0.84 ± 0.05op	0.08 ± 0.02f–l
		24	0.13 ± 0.001d–h	0.23 ± 0.01k–p	0.94 ± 0.03n–p	0.07 ± 0.01i–m
		48	0.11 ± 0.0003g–k	0.34 ± 0.01a–c	1.41 ± 0.07f–j	0.11 ± 0.01a–e
		72	0.11 ± 0.01f–j	0.38 ± 0.003ab	1.25 ± 0.28i–m	0.12 ± 0.01a—-d
Medium	5	0	0.15 ± 0.01c–e	0.28 ± 0.01d–k	1.11 ± 0.04l–n	0.09 ± 0.01c–i
		24	0.13 ± 0.01d–h	0.22 ± 0.005l–p	1.24 ± 0.12j–m	0.07 ± 0.01i–m
		48	0.16 ± 0.005b–d	0.25 ± 0.005h–n	1.46 ± 0.07e–h	0.11 ± 0.0001b–f
		72	0.15 ± 0.003c–e	0.26 ± 0.01g–n	0.91 ± 0.01n–p	0.08 ± 0.01g–l
	10	0	0.15 ± 0.01c–e	0.28 ± 0.01d–k	1.11 ± 0.04l–n	0.09 ± 0.01c–i
		24	0.09 ± 0.004jk	0.25 ± 0.001i–n	1.18 ± 0.05k–m	0.10 ± 0.001b–g
		48	0.11 ± 0.004f–j	0.32 ± 0.02c–f	1.42 ± 0.07 e–j	0.10 ± 0.01b–g
		72	0.12 ± 0.01f–j	0.18 ± 0.02pq	0.96 ± 0.07n–p	0.08 ± 0.02f–l
	15	0	0.15 ± 0.01c–e	0.28 ± 0.01d–k	1.11 ± 0.04l–n	0.09 ± 0.01c–i
		24	0.09 ± 0.004jk	0.27 ± 0.002f–l	1.37 ± 0.04f–k	0.09 ± 0.005e–j
		48	0.12 ± 0.007f–j	0.19 ± 0.01o–q	1.92 ± 0.04ab	0.07 ± 0.002j–m
		72	0.10 ± 0.01jk	0.23 ± 0.01k–p	1.40 ± 0.11f–j	0.09 ± 0.01d–j
High	5	0	0.18 ± 0.01bc	0.33 ± 0.02b–d	1.70 ± 0.11c–e	0.11 ± 0.02a–e
		24	0.12 ± 0.004f–j	0.26 ± 0.01g–m	1.46 ± 0.16e–i	0.07 ± 0.003i–m
		48	0.15 ± 0.01de	0.27 ± 0.005f–l	1.58 ± 0.07c–g	0.11 ± 0.01a–e
		72	0.11 ± 0.02f–j	0.30 ± 0.01c–j	1.25 ± 0.10h–l	0.09 ± 0.01e–k
	10	0	0.18 ± 0.04bc	0.33 ± 0.02b–d	1.70 ± 0.11c–e	0.11 ± 0.023a–e
		24	0.15 ± 0.01de	0.28 ± 0.005e–k	1.71 ± 0.10b–d	0.10 ± 0.01b–f
		48	0.10 ± 0.0004jk	0.28 ± 0.07d–k	2.05 ± 0.07a	0.16 ± 0.003a
		72	0.12 ± 0.02f–j	0.26 ± 0.02g–n	1.63 ± 0.03c–f	0.13 ± 0.001ab
	15	0	0.18 ± 0.01bc	0.33 ± 0.01b–d	1.70 ± 0.11c–e	0.11 ± 0.02a–e
		24	0.13 ± 0.003d–h	0.33 ± 0.005b–e	1.49 ± 0.08d–h	0.09 ± 0.01e–j
		48	0.16 ± 0.01b–e	0.38 ± 0.01ab	1.99 ± 0.04a	0.12 ± 0.01abc
		72	0.19 ± 0.001b	0.40 ± 0.004a	1. 77 ± 0.06 bc	0.14 ± 0.001ab

Mean g/Kg ± standard deviation (*n* = 3). TGI, tetragalloyl glucose isomer; EAP, ellagic acid pentoside; AGN, agrimoniin; EA, free ellagic acid. Different letter in a column indicates significantly differences among all treatments.

**TABLE 1B:** Changes in the individual phenolic compound concentration of strawberry by–products as affected by UVA dose, storage time, and temperature.

UVA	Storage temp (°C)	Storage time (h)	PACT (g/Kg)	Q3G (g/Kg)	K3G (g/Kg)	KP (g/Kg)	TPC_HPLC_ (g/Kg)
RF-N	5	0	0.19 ± 0.01a	0.18 ± 0.01j–n	0.10 ± 0.01f–h	0.01 ± 0.003h–l	1.71 ± 0.10o–q
		24	0.10 ± 0.0001i–q	0.18 ± 0.03k–o	0.11 ± 0.02d–f	0.01 ± 0.003 j–l	1.76 ± 0.32o–q
		48	0.12 ± 0.05 f–p	0.20 ± 0.005e–k	0.11 ± 0.004c–f	0.01 ± 0.001e–j	1.88 ± 0.32m–p
		72	0.10 ± 0.001j–q	0.19 ± 0.04 h–m	0.10 ± 0.02f–j	0.01 ± 0.004h–l	1.66 ± 0.06pq
	10	0	0.19 ± 0.01a	0.18 ± 0.01j–n	0.10 ± 0.01f–h	0.01 ± 0.004i–l	1.71 ± 0.10o–q
		24	0.12 ± 0.01f–o	0.19 ± 0.03 h–m	0.11 ± 0.02d–f	0.01 ± 0.002kl	2.16 ± 0.38j–m
		48	0.11 ± 0.003 h–p	0.17 ± 0.002 k–o	0.11 ± 0.004c–f	0.01 ± 0.002j–l	1.97 ± 0.04k–o
		72	0.12 ± 0.004 f–o	0.16 ± 0.0002m–o	0.08 ± 0.01ij	0.002 ± 0.0001m	1.53 ± 0.21q
	15	0	0.19 ± 0.01a	0.18 ± 0.01j–n	0.10 ± 0.01f–h	0.01 ± 0.0004h–l	1.71 ± 0.10o–q
		24	0.12 ± 0.003f–n	0.16 ± 0.005 l–o	0.08 ± 0.01 h–j	0.01 ± 0.0001l	1.69 ± 0.18o–q
		48	0.14 ± 0.02d–i	0.19 ± 0.001h–n	0.10 ± 0.002 f–h	0.01 ± 0.0004h–l	2.26 ± 0.17g–k
		72	0.09 ± 0.03k–q	0.18 ± 0.05j–n	0.10 ± 0.03f–i	0.01 ± 0.004i–l	2.21 ± 0.31h–k
Low	5	0	0.13 ± 0.03e–k	0.18 ± 0.01i–n	0.10 ± 0.01f–h	0.01 ± 0.001g–l	1.80 ± 0.10o–q
		24	0.12 ± 0.04f–n	0.21 ± 0.004d–j	0.11 ± 0.004d–f	0.02 ± 0.001d–i	2.33 ± 0.05f–j
		48	0.06 ± 0.001qr	0.20 ± 0.02e–k	0.11 ± 0.01d–f	0.02 ± 0.002c–g	2.65 ± 0.13c–e
		72	0.09 ± 0.04m–r	0.09 ± 0.020g–m	0.10 ± 0.01f–h	0.01 ± 0.001i–l	1.86 ± 0.16 n–p
	10	0	0.13 ± 0.03e–k	0.18 ± 0.01i–n	0.10 ± 0.01f–h	0.01 ± 0.001g–l	1.80 ± 0.10o–q
		24	0.08 ± 0.03o–r	0.14 ± 0.06o	0.07 ± 0.03j	0.01 ± 0.004l	1.81 ± 0.25 o–q
		48	0.14 ± 0.003c–g	0.16 ± 0.001m–o	0.07 ± 0.02j	0.01 ± 0.0002i–l	2.25 ± 0.03g–k
		72	0.05 ± 0.003r	0.14 ± 0.0004o	0.09 ± 0.001g–j	0.01 ± 0.001l	2.37 ± 0.07e–j
	15	0	0.13 ± 0.03e–k	0.18 ± 0.01i–n	0.10 ± 0.01f–h	0.01 ± 0.001g–l	1.80 ± 0.10o–q
		24	0.13 ± 0.01e–k	0.18 ± 0.02h–n	0.09 ± 0.01f–j	0.01 ± 0.002l	1.84 ± 0.02n–p
		48	0.11 ± 0.05g–p	0.26 ± 0.005a–c	0.13 ± 0.001b–e	0.02 ± 0.003a–c	2.53 ± 0.14d–g
		72	0.12 ± 0.01f–m	0.29 ± 0.001a	0.15 ± 0.003ab	0.02 ± 0.0002ab	2.53 ± 0.31d–g
Medium	5	0	0.17 ± 0.0001a–d	0.20 ± 0.01f–k	0.11 ± 0.01e–g	0.01 ± 0.004d–i	2.21 ± 0.05h–k
		24	0.13 ± 0.01f–l	0.18 ± 0.001j–n	0.09 ± 0.002 f–j	0.01 ± 0.0003l	2.14 ± 0.14 j–n
		48	0.13 ± 0.0001e–k	0.22 ± 0.01d–h	0.13 ± 0.02a–d	0.01 ± 0.0002h–l	2.53 ± 0.09d–g
		72	0.13 ± 0.03f–k	0.19 ± 0.01g–l	0.10 ± 0.0001f–i	0.01 ± 0.002g–l	1.90 ± 0.07l–p
	10	0	0.17 ± 0.0001a–d	0.20 ± 0.01f–k	0.11 ± 0.01e–g	0.02 ± 0.004d–i	2.21 ± 0.05h–k
		24	0.18 ± 0.002a–c	0.18 ± 0.02h–n	0.10 ± 0.001f–i	0.01 ± 0.006l	2.18 ± 0.05i–l
		48	0.11 ± 0.03h–p	0.22 ± 0.012c–g	0.13 ± 0.01b–e	0.02 ± 0.006 b–f	2.49 ± 0.15 d–h
		72	0.09 ± 0.01n–r	0.21 ± 0.02d–j	0.10 ± 0.01f–i	0.01 ± 0.001kl	1.80 ± 0.12o–q
	15	0	0.17 ± 0.0001a–d	0.20 ± 0.01f–k	0.11 ± 0.01e–g	0.02 ± 0.005d–i	2.21 ± 0.05h–k
		24	0.08 ± 0.002p–r	0.17 ± 0.01k–o	0.10 ± 0.004f–j	0.01 ± 0.0001f–k	2.24 ± 0.06g–k
		48	0.12 ± 0.001g–p	0.15 ± 0.01no	0.08 ± 0.02g–j	0.01 ± 0.005j–l	2.71 ± 0.09cd
		72	0.13 ± 0.01d–j	0.18 ± 0.01j–n	0.14 ± 0.005ab	0.01 ± 0.001j–l	2.33 ± 0.04f–j
High	5	0	0.14 ± 0.02c–h	0.24 ± 0.001b–d	0.13 ± 0.01a–c	0.01 ± 0.003 a–d	2.91 ± 0.17bc
		24	0.17 ± 0.0004a–d	0.19 ± 0.003 h–m	0.10 ± 0.004f–i	0.01 ± 0.001f–k	2.47 ± 0.16d–i
		48	0.19 ± 0.01ab	0.20 ± 0.14 f–k	0.14 ± 0.0003ab	0.002 ± 0.001a–d	2.74 ± 0.09cd
		72	0.17 ± 0.01a–e	0.24 ± 0.14 b–e	0.14 ± 0.005 a–c	0.02 ± 0.0015c–g	2.37 ± 0.08e–j
	10	0	0.14 ± 0.01c–h	0.24 ± 0.001b–d	0.13 ± 0.01a–c	0.02 ± 0.003 a–d	2.91 ± 0.17bc
		24	0.13 ± 0.003c–h	0.18 ± 0.01k–o	0.15 ± 0.01a	0.01 ± 0.0003f–k	2.76 ± 0.09cd
		48	0.19 ± 0.003ab	0.22 ± 0.01c–g	0.11 ± 0.02e–g	0.02 ± 0.001a–d	3.21 ± 0.01a
		72	0.12 ± 0.02l–r	0.22 ± 0.01d–i	0.11 ± 0.02e–g	0.02 ± 0.0001a–e	2.62 ± 0.06 c–f
	15	0	0.14 ± 0.02c–h	0.24 ± 0.001b–d	0.13 ± 0.01a–c	0.02 ± 0.0003 a–d	2.91 ± 0.2bc
		24	0.10 ± 0.003i–q	0.23 ± 0.005c–f	0.14 ± 0.01ab	0.02 ± 0.0001c–h	2.57 ± 0.08d–f
		48	0.16 ± 0.003b–f	0.24 ± 0.005b–d	0.15 ± 0.01ab	0.02 ± 0.001a	3.30 ± 0.06a
		72	0.15 ± 0.01c–g	0.27 ± 0.002ab	0.13 ± 0.01a–e	0.02 ± 0.001ab	3.10 ± 0.07ab

Mean g/Kg ± standard deviation (*n* = 3). PACT, procyanidin tetramer; Q3, quercetin 3-o-glucuronide; K3G, Kaempferol 3-o-glucuronide; TPC_HPLC_, Total phenolic content by HPLC.

Different letter in a column indicates significantly differences (*p* < 0.05) between all treatments studied.

**FIGURE 4:**
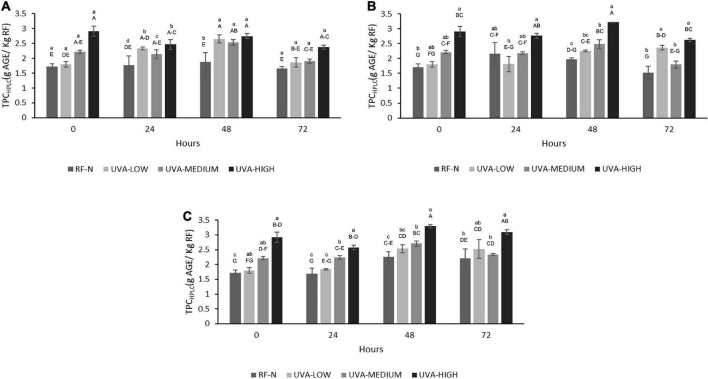
Total phenolic content determined by HPLC (TPC_HPLC_) of UVA-treated strawberry by-products during storage at **(A)** 5°C, **(B)** 10°C, **(C)** 15°C. RF-N: Strawberry agro-industrial by-product control. UVA-LOW: RF irradiated with 28.8 KJ/m^2^. UVA-MEDIUM: RF irradiated with 57.6 KJ/m^2^. UVA-HIGH: RF irradiated with 86.4 KJ/m^2^. Different lower-case letters, mean significant differences (*p* ≤ 0.05) among different treatments at the same storage time. Different capital letters mean significant differences (*p* ≤ 0.05) among different storage times.

After exposure of RF to the different UVA doses, the TPC_HPLC_ changed similarly to the TPC determined by the Folin-Ciocalteu method, with no significant differences between the control and UVA-LOW samples (1.7–1.8 g/Kg). However, for UVA-MEDIUM, the phenolic compounds increased by 29%, and the most significant increase was observed in the UVA-HIGH treatment (2.9 g/Kg), which was 70% higher than the RF control. For storage at 5°C, the highest increases occurred after 24–48 h with an average content of 2.5 g/kg (Figure 4). There were no significant differences among TPC_HPLC_ of RF-E samples after 24–48 h at 5°C, being up to 54% higher than the control for the same period, and similar to that reported by Surjadinata et al. (8), who applied UVA 12.7 Wm^–2^ for 6 h on wounded carrots, achieving a 100% increase in total phenols and up to 300% increase in chlorogenic acid. At 10°C, the highest increases were determined after 24–48 h with an average content of 2.5 g/kg. However, the highest accumulation of phenolic compounds was obtained after 48 h at 15°C (3.3 g/Kg RF), 104% higher than the control (Figure 4). Similar to the results obtained for broccoli by-products using UVB-C 10 KJ/m^2^, after 48 h at 15°C showed an increase of 88% of phenolic compounds (38).

**FIGURE 5:**
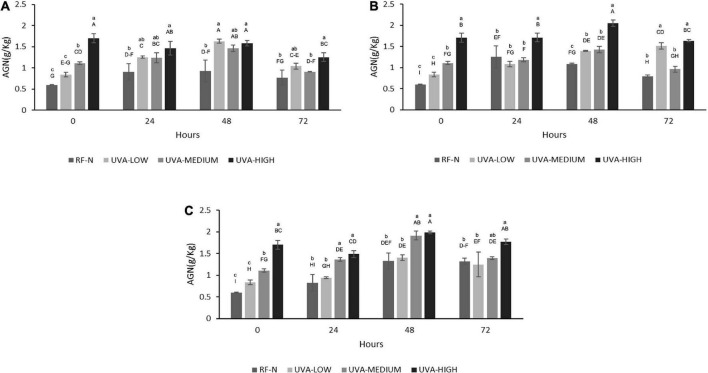
Agrimoniin concentration (AGN) of UVA-treated strawberry by-products during storage at **(A)** 5°C, **(B)** 10°C, **(C)** 15°C. RF-N: Strawberry agro-industrial by-product control. UVA-LOW: RF irradiated with 28.8 KJ/m^2^. UVA-MEDIUM: RF irradiated with 57.6 KJ/m^2^. UVA-HIGH: RF irradiated with 86.4 KJ/m^2^. Different lower-case letters mean significant (*p* ≤ 0.05) differences among treatments at the same storage time. Different capital letters mean significant differences (*p* ≤ 0.05) among different storage times.

The results in Figure 5 indicate the positive effect of UVA on the biofortification of strawberry agro-industrial by-products with AGN. The initial accumulation of AGN was proportional to the UVA dose being UVA-LOW < UVA-MEDIUM < UVA-HIGH reaching an increase of 184% (1.7 g/Kg) compared to the control (0.6 g/Kg). A maximum accumulation of AGN (2.0 g/kg) was achieved during storage at 10°C for 48 h, obtaining a higher increase than that obtained by Moreira-Rodríguez et al. (34) in broccoli sprouts applying UVA radiation (3.16 W/m^2^ for 120 min), achieving a maximum increase in the compound 5-sinapoyl-quinic acid (121%). As earlier described, although the mechanism of how UVA modulates the biosynthesis of phenolic compounds is not fully elucidated, these results show AGN as the compound with the highest accumulation in RF, which is in contrast with previous reports for fruit subjected to pre-harvest UVB radiation, where anthocyanins and flavonols are mainly increased (42). In addition, UVA radiation is shown to be an efficient non-thermal alternative technology to biofortify with phenolic compounds and strawberry agro-industrial conditioning by-products.

### Determination of the optimum biofortification process conditions to increase phenolics accumulation in the strawberry by-product

Based on Derringer’s desirability function, the multiple response optimization procedure was applied to determine the optimal experimental conditions of the biofortification in RF phenolic compounds. The objective was to obtain the optimum UVA dose, storage time (ST), and temperature (TM) that maximize the content of phenolic compounds (TPC and TPC_HPLC_), agrimoniin content (AGN), and PAL activity, minimizing PPO activity in the strawberry by-products.

Table 2 shows the results from the simultaneous optimization. The optimum biofortification process conditions to increase phenolics accumulation in the strawberry by-products were a UVA dose of 86.4 KJ/m^2^ (UVA-HIGH), ST = 15°C, and TM = 46 h. Under these conditions, the predicted values of the different responses were: PAL = 0.13 ΔA mg protein^–1^h^–1^, PPO = 0.16 ΔA mg protein^–1^h^–1^, TPC, 16.7 g AGE/Kg RF, TPC_HPLC_ = 3.1 g/Kg RF and, AGN = 1.9 g/Kg RF (Table 3). Finally, the biofortification process was carried out at the optimal conditions to validate the predicted results and to determine the antioxidant and anti-inflammatory properties of UVA-induced biofortification phenolic compounds. The experimental results obtained are shown in Table 2. The experimental values obtained for TPC, TPC_HPLC_, and AGN were similar (*p* > 0.05) to the predicted values, indicating the models’ adequacy. However, the experimental PAL values were higher (23%) than the predicted values, while the experimental PPO values were lower (25%) than the predicted values.

**TABLE 2:** Optimal biofortification treatment of strawberry by-products (RF) and their experimental and predicted response values.

Optimized treatment variables			Responses	PAL (ΔA mg protein^–1^ h^–1^)	PPO (ΔA mg protein^–1^ h^–1^)	TPC (g AGE/Kg)	TPC_HPLC_ (g AGE/Kg)	AGN (g/Kg)

UVA	ST (°C)	TM (h)						
High	15	46	*Predicted*	0.13b	0.16a	16.7a	3.1a	1.9a
High	15	46	*Experimental*	0.16a	0.12b	15.9a	2.9a	1.7a

UVA, UVA radiation dose; ST, storage temperature; TM, storage time; PAL, Phenylalanine Ammonia Lyase activity; PPO, Polyphenol Oxidase activity; TPC, Total phenolic content; TPC_HPLC_, Total phenolic content by HPLC; AGN, Agrimoniin. Different letter indicates significant differences between Predicted and Experimental values *p* < 0.05 by t-test.

**TABLE 3:** Bioaccessibility of strawberry by-products (RF) phenolic compounds.

Sample	Compound	Salival fluid	Gastric phase	Intestinal phase	Bioaccessibility (%)	
			
		g/Kg			Gastric phase	Instestinal phase
RF-N	TGI	0.10 ± 0.003a	0.05 ± 0.001b	0.07 ± 0.001ab*	49.8	71.8*
	EAP	0.34 ± 0.005a	0.04 ± 0.001b	0.02 ± 0.001c	11.8	7.1
	AGN	1.35 ± 0.06a	0.15 ± 0.01b	0.13 ± 0.004b	10.9	9.80
	EA	0.13 ± 0.003a	0.03 ± 0.005b	0.01 ± 0.0001c	19.9	9.1
	PACT	0.14 ± 0.01a	0.11 ± 0.002b	0.11 ± 0.01b	79.2	79.2
	Q3G	0.23 ± 0.01a	0.05 ± 0.001b	0.02 ± 0.001c	19.9	10.0
	K3G	0.23 ± 0,01a	0.04 ± 0.002b	0.02 ± 0.001c	16.2	6.8
	KP	0.02 ± 0.001	n.d	n.d	n.d	n.d
	TPC_HPLC_	2.55 ± 0.07a	0.46 ± 0.02b	0.39 ± 0.003b	18.0	15.3
RF-E	TGI	0.15 ± 0.004a*	0.08 ± 0.03b	0.05 ± 0.003b	51.4	36.1
	EAP	0.36 ± 0.002a*	0.10 ± 0.02b*	0.04 ± 0.004c*	27.2*	11.1*
	AGN	1.79 ± 0.03a*	0.51 ± 0.01b*	0.45 ± 0.01c*	28.4*	25.1*
	EA	0.15 ± 0.004a*	0.04 ± 0.002b*	0.03 ± 0.002c*	28.1*	17.9*
	PACT	0.17 ± 0.01a*	0.12 ± 0.003b*	0.10 ± 0.01b	71.7*	59.7
	Q3G	0.26 ± 0.01a*	0.07 ± 0.004b*	0.03 ± 0.001c*	27.2*	11.9*
	K3G	0.28 ± 0.01a*	0.05 ± 0,002b*	0.02 ± 0.005c	19.0*	5.7
	KP	0.04 ± 0.001*	n.d	n.d	n.d	n.d
	TPC_HPLC_	3.20 ± 0.01a*	0.97 ± 0.04b*	0.71 ± 0.001c*	30.3*	22.2*

RF-N, Strawberry by-product control; RF-E, Strawberry by-product under optimal biofortification conditions TGI, tetragalloyl glucose isomer (TGI); EAP, ellagic acid pentoside; AGN, agrimoniin; EA, free ellagic acid. procyanidin tetramer; Q3, quercetin 3-o-glucuronide; K3G, Kaempferol 3-o-glucuronide; TPC_HPLC_, Total phenolic content by HPLC. n.d, Not detected.

Different lower-case letters in the same row indicate significant differences by Tukey’s test (*p* < 0.05). *: Means differences (*p* < 0.05) between the same compound of RF-N and RF-E samples.

### *In vitro* gastrointestinal digestion assay

The beneficial health effects of phenolic compounds depend not only on the profile or amount of compounds but also on the release and transformation during the physiological processes of digestion. However, the low bioaccessibility of phenolic compounds has been widely reported due to the biotransformations they undergo during the digestive process (43). As RFs are an important source of phenolic compounds, it is important to know their behavior and stability during the gastric process, as these by-products can be revalued by obtaining dietary supplements, and it is necessary to guarantee an adequate and safe bioaccessibility of the bioactive compounds. The individual phenolic compounds were quantified at the different stages of the *in vitro* digestion test. The results are shown in Table 3, where the RF-N and RF-E phenolic bioaccessibility was compared. The different phases showed a significant effect (*p* < 0.05) on the concentration of individual phenolic compounds (EAP, EA, Q3G, and K3G) for RF-N and RF-E (EAP, AGN, EA, Q3G, K3G, TPC_HPLC_). AGN ellagitannin was the major compound in all intestinal phases for both RF-N and RF-E, showing a decrease in initial concentration from 1.35 g/kg in RF-N to 0.13 g/kg in the IF, having a bioaccessibility of 9.8%. RF-E started with 1.79 g/kg, and the digestive process reduced it to 0.45 g/kg in IF (25.1%). These results agree with other reports regarding the digestive stability of phenolic compounds from different plant matrices (43–45).

Concerning the bioaccessibility of phenolics from strawberries, it has been reported that anthocyanins, their primary group of phenolic compounds, present low bioaccessibility (1% on average) (43, 46). Ariza et al. (47) reported the bioaccessibility of strawberry achenes which were the majority and the most bioaccessible, with 0.0297 g/Kg of hydrolyzable tannins in the intestinal phase. On the other hand, the PACT compound had the highest bioaccessibility in the intestinal phase, with 79.2% for RF-N and 59.7% for RF-E. These results align with those reported for epicatechin bioaccessibility in sweet cherry with 40% and those reported for cocoa procyanidins with a range of 68.5–70.9% (48, 49). Thus, showing the importance of procyanidins as a source of bioactive compounds with high bioaccessibility. This was followed by the TGI with 71.8 and 36.1%, respectively, indicating that the biofortification process with UVA reduced the bioaccessibility of this compound. K3G was the compound with the lowest bioaccessibility in RF-N (6.8%) and RF-E (5.7%). The phenolic compounds of the different parts of strawberries are not very stable at the basic pH typical of the gastric phase. However, compounds such as ellagic acid in RF-E showed a bioaccessibility of 24% in the gastric phase and 17.9% in the intestinal phase, similar to that reported for blackberry with 14 % (43). Quercetin and kaempferol glycosides were the compounds most affected by the digestive process, as gastric pH generates the biotransformation of these compounds into aglycones which are more unstable. These observations have already been reported on the bioaccessibility of whole strawberries (45).

Regarding TPC_HPLC_, the trend of decreasing content in the different stages of digestion continues, initially with a content of 2.5 and 3.2 g/Kg for RF-N and RF-E, respectively; they reached the intestinal phase with 0.39 and 0.71 g/Kg, respectively. According to Olivero-David et al. (50), about 95% of phenolic compounds that reach the intestinal phase pass directly to the large intestine without being absorbed, interacting with the microbiota, and through their enzyme complexes, new metabolites with different bioactivities are obtained. It was also observed that the biofortification process using UVA radiation and subsequent storage at 15°C for 46 h significantly improved the content of phenolic compounds that reached the intestinal phase (EAP, AGN, PACT, Q3G, and TPC_HPLC_).

### Antiproliferative activity of radiation to strawberry agro-industrial by-products digested fraction on healthy control and cancer cells

**FIGURE 6:**
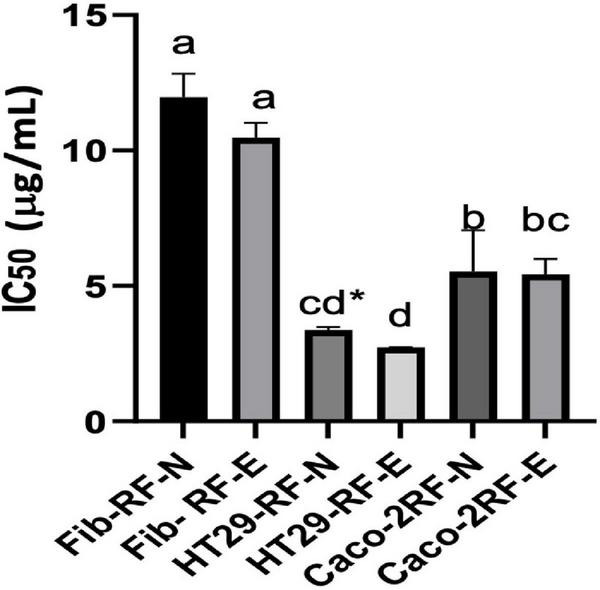
IC_50_ determination on HDFa and Cancer cell lines. Fib: Adult primary dermal fibroblast cell line. HT29: Human Colorectal Adenocarcinoma cell line. Caco-2: Human colorectal adenocarcinoma cell line. Different letter indicates significant differences between IC_50_ of different cell lines *p* < 0.05 by Tukey’s test. * Means significantly differences (*p* < 0.05) between strawberry by-product control (RF-N) and: Strawberry by-product biofortified (RF-E) according to *t*-test *p* < 0.05.

There is little information on the anticancer potential of strawberry agro-industrial residues. In addition, RF, being a rich source of phenolic compounds, during the digestive process when consuming these compounds from RF, have a contact time in the colon, thus in this work, we evaluated the antiproliferative capacity in colon cancer lines (Caco-2 and HT29) compared to a control with healthy HDFA cell lines (20). Figure 6 shows the IC_50_ determined for the digested extract biofortified with phenolic compounds under optimal conditions (RF-E) compared to the digested control extract (RF-N) in colon cancer cells, and as healthy control cells, the HDFa cell line (Fib) was used. A dose-dependence against the proliferation of cell viability was evident in all 3 cell lines. For Caco-2, both extracts showed an IC_50_ of 5.53 and 5.43 μg/mL for RF-N and RF-E, respectively. However, for HT29, the IC_50_ of the RF-E extract (2.73 μg/mL) was significantly lower than RF-N (3.37 μg/mL). Compared to the control with healthy cells (Fib) with 11.97–10.47 μg/mL for RF-N and RF-E, respectively, a significantly higher IC_50_ was observed compared to the IC_50_ of the cancer lines studied, being lower than the IC_50_ reported for blueberry phenolic compounds in Caco-2 and HT29 cells (15–50 μg/mL) (51).

According to the results in Table 3, in the RF-E extracts, the main phenolic compound in the digested fraction was agrimoniin, a dimer composed of two α-1-O-galloyl-2,3:4,6-bis-hexahydroxydiphenoylD-glucose units linked by a C-O-C bond between two gallic acid residues. In some berries, the digestive process enhances the antiproliferative activity of quercetin and kaempferol glycosides (52); however, for RF, the release of flavonol aglycones negatively affected the stability of these compounds. Several authors have validated the antiproliferative effect of strawberry phenolic compounds (52–54) with an average IC_50_ for HT29 of 114 μg/mL; however, there is no known report on the antiproliferative activity of phenolic compounds from strawberry agro-industrial residues, which showed better activity than whole fruit compounds with a lower IC_50_. These results show the antiproliferative potential of the strawberry agro-industrial by-product on colon cancer cells to be more significant in the biofortified extract with UVA application and safe in healthy cells. Since colorectal cancer is the primary type of cancer and has one of the highest mortality rates in society, it is important to revalue strawberry agro-industrial by-products through biofortification with phenolic compounds, to extract the compounds and use the extract to prevent this disease.

### Cellular antioxidant activity

**FIGURE 7:**
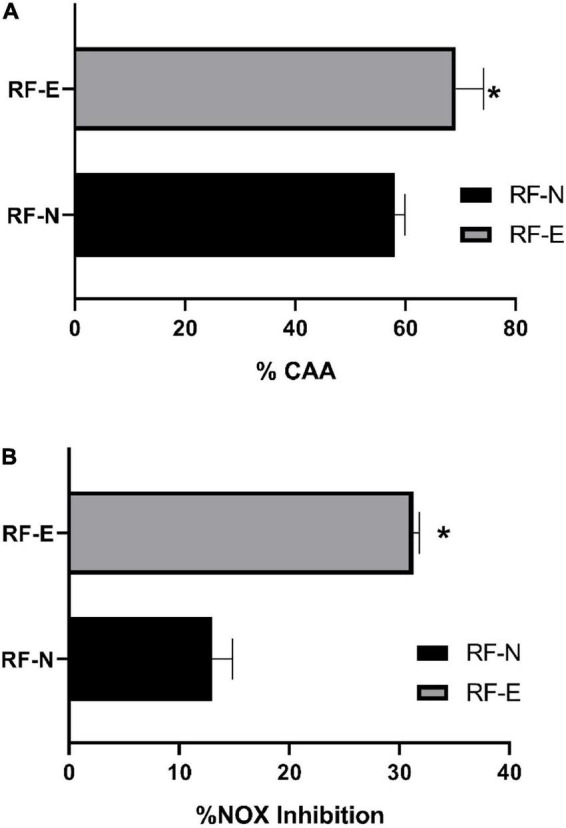
**(A)** Cellular antioxidant capacity inhibition (%CAA) by APPH radical. **(B)** Nitric Oxide (NOX) cell production inhibition (%). Bar indicates standard deviation. *: Means significantly differences between strawberry by-product control (RF-N) and strawberry by-product biofortified (RF-E) samples, according to *t*-test *p* < 0.05.

The by-products of strawberry processing have ellagitannins as the main compounds, which, depending on their degree of polymerization, have better bioactivity, as their amphiphilic character allows them to diffuse passively into the cell membrane and then pass into the aqueous cell medium (55). Imbalances between pro-oxidant and antioxidant species at the physiological level trigger several conditions that facilitate the development of chronic non-communicable diseases (6). Figure 7A shows the antioxidant cellular capacity of digested RF extracts (2.2 mg/mL) to inhibit the APPH radical on the Caco-2 cell line. The RF-E showed a significantly higher antioxidant capacity (69%) than the digested extract of RF-N (58 %), being 11% higher, thanks to the content of phenolic compounds that reach bioaccessibility in the biofortified tissue, being higher than that reported by Lang (56) for the blueberry extract with 25% CAA.

According to the phenolic profile identified in the digested fraction of the RF-E, ellagitannins were the major compounds that have been widely reported in the different parts of the strawberry plant, where, in addition, the *in vitro* free radical inhibitory capacity has been demonstrated (2). Oxidases are one of the primary sources of ROS in the cell. The enzymes nitric oxide synthase and nitric oxide synthase produce nitric oxide from L-arginine and molecular oxygen. Triggering an overproduction of ROS, ellagitannins of RF-digested extracts with their hydroxyl groups can neutralize free radicals, especially agrimoniin, which has been reported as a potent free radical scavenger (57). Regular strawberry consumption has recently been reported to reduce ROS production and oxidative biomarker levels (58, 59). However, little information is available on the cellular antioxidant potential of the various parts of the strawberry plant, such as the sepal, stalk, and leaves that constitute the residue of agro-industrial strawberry conditioning. This work demonstrated the CAA potential of these by-products, allowing them to be used in the search for sustainable alternatives for obtaining bioactive compounds.

### Anti-inflammatory potential

The process of chronic inflammation is correlated with the development of metabolic syndrome, which has become one of the major causes of death in the Western world due to poor lifestyle habits (60). Therefore, compounds with an effective anti-inflammatory activity that prevents the development of a pre-disease state into a chronic disease are currently being sought. The anti-inflammatory potential of phenolic compounds in the RAW cell line exhibits a dose-dependent behavior. In this study, the concentration at which the viability of RAW cells was not significantly affected (>80%) was determined at a concentration of 1.1 mg/mL Figure 7B shows the nitric oxide inhibition capacity in RAW 264.7 cells. The RF-N digested tissue with a concentration of 1.1 mg/mL achieved 13% inhibition of NOX production, while RF-E was significantly higher (*p* < 0.05) with 31% inhibition. Fang et al. (61) reported for purified oxyresveratrol extracts with a concentration of 6 μg/mL a 50% reduction in NOX production, compared to the digested crude extract of RF-E, which achieved 31%, is promising as the unpurified extract has good anti-inflammatory activity. Fumagalli et al. (62) studied digested strawberry extract (10 μg/mL), which had tannins as major compounds, and found a potent inhibition of IL-8 secretion, and procyanidins showed prevention of TNF-α induction. Ellagitannins and their metabolites can control inflammatory mediators such as TNF-α, IL-1β, IL-6, and IL-8 by inhibiting oxidase enzymes (NOS) (63). According to Table 3, agrimoniin was the main compound after the RF digestion process and is probably the compound with the highest bioactivity in RF extracts. Moreover, Hoffmann et al. (64) reported that agrimoniin-rich fractions of *Potentilla erecta* showed high anti-inflammatory effects in *in vitro* and *in vivo* assays. Biofortified agro-industrial by-products have a relevant bioactive potential that allows a greater revalorization of these by-products by obtaining extracts rich in high-value hydrolyzable tannins such as agrimoniin.

## Conclusion

Agro-industrial strawberry residues responded to the elicitor effect of UVA radiation and storage at different temperatures activating PAL activity, PPO, and *de novo* synthesis of phenolic compounds. In addition, the biofortification process was successfully optimized. The optimum biofortification point was applying a UVA-HIGH (86 KJ/m^2^) and then storing at 15°C for 46 h. The use of UVA radiation and storage as a tool for biofortification with phenolic compounds for the revalorization of strawberry agro-industrial by-products proved to be an alternative technology that effectively induced the secondary metabolism of these waste tissues, obtaining increases of 184% concerning the control. Agrimoniin (AGN) was the compound mainly generated by applying UVA radiation (1.9 g/Kg RF) with 25% of bioaccessibility. Procyanidin was the compound with the highest bioaccessibility with 59.7%. The biofortification process applied significantly improved the bioaccessibility of the total phenolic compounds and their bioactivities, such as antiproliferative capacity in Caco-2 and HT29 colorectal cancer cells, and the safety of RF extract demonstrated on the HDFa (Fib) cell line, as the IC_50s_ for Fib were significantly higher (10 μg/mL) than those shown for Caco2 and HT29 (5.43–2.73 μg/mL, respectively) and cellular antioxidant capacity, and anti-inflammatory activity, where RF-E (69 and 31%, respectively) showed superiority over RF-N (58 and 13%, respectively). Thus, applying UVA radiation in strawberry by-products allowed a greater revalorization of RF through increasing phenolic compound content and bioactivity, turning this by-product into a sustainable, low-cost source of valuable bioactive compounds with preventive applications in chronic degenerative diseases.

## Data availability statement

The raw data supporting the conclusions of this article will be made available by the authors, without undue reservation.

## Author contributions

EV-G: conceptualization, data curation, validation, investigation, writing – original draft, and formal analysis. MA-R: validation, investigation, methodology, and writing. AP: conceptualization, reviewing and editing, supervision, project administration, and funding acquisition. DJ-V: conceptualization, methodology, writing – reviewing and editing, supervision, project administration, and funding acquisition. All authors contributed to the article and approved the submitted version.
