# Supplementary material for: Adding value to strawberry agro-industrial by-products through ultraviolet A-induced biofortification of antioxidant and anti-inflammatory phenolic compounds
Source: Front Nutr. 2022 Dec 7;9:1080147. doi: 10.3389/fnut.2022.1080147 (PMC9769405; doi:10.3389/fnut.2022.1080147)
Supplement: Supplementary file 1 [file Data_Sheet_1.docx]

Supplementary Material

Adding value to strawberry agro-industrial by-products through UVA induced biofortification of antioxidant and anti-inflammatory phenolic compounds

**Esteban Villamil-Galindo^1,2^, Marilena Antunes-Ricardo^3,4^, Andrea Marcela Piagentini^1^, Daniel A. Jacobo-Velázquez^5,6*^**

^1^Instituto de Tecnología de Alimentos, Facultad de Ingeniería Química, Universidad Nacional del Litoral, Santa Fe, Argentina.

^2^Consejo Nacional de Investigaciones Científicas y Técnicas (CONICET), Santa Fe, Argentina.

^3^Tecnologico de Monterrey, The Institute for Obesity Research, Monterrey, México.

^4^Tecnologico de Monterrey, Escuela de Ingeniería y Ciencias, Monterrey, México.

^5^Tecnologico de Monterrey, The Institute for Obesity Research, Zapopan, México.

^6^Tecnologico de Monterrey, Escuela de Ingeniería y Ciencias, Zapopan, México.

*** Correspondence:**Daniel A. Jacobo-Velázquez
djacobov@tec.mx

**Table S-1:** Compositions of simulated gastrointestinal phases

| Reactive | Salival fluid | Gastric phase | Intestinal Phase | Duodenal Phase |
| --- | --- | --- | --- | --- |
| Deionized water (mL) | 500 | 500 | 500 | 500 |
| NaCl (g) | 0.0585 | 2.752 | 7.012 | 5.259 |
| KCl (g) | 0.0745 | 0.824 | 0.564 | 0.376 |
| NaHCO_3_ (g) | 1.05 |  | 3.388 | 5.785 |
| Urea (g) | 0.2 | 0.085 | 0.1 | 0.25 |
| NaH_2_PO_4_ (g) |  | 0.266 |  |  |
| CaCl._2_H_2_O (g) |  | 0.399 |  |  |
| NH_4_Cl (g) |  | 0.306 |  |  |
| HCl (mL) |  | 6.5 | 0.18 | 0.15 |
| KH_2_PO_4_ (g) |  |  | 0.08 |  |
| MgCl_2_ (g) |  |  | 0.05 |  |
| Enzymes |  |  |  |  |
| α-Amylase (g) | 1 |  |  |  |
| Pepsin (g) |  | 2.5 |  |  |
| Pancreatin (g) |  |  | 9 |  |
| Lipase (g) |  |  | 1.5 |  |
| Bile Salt (g) |  |  |  | 8.2 |

**Table S-2:** ANOVA for the Phenylalanine Ammonia Lyase (PAL), Polyphenol oxidase (PPO) activity, Total phenolic content (TPC), Total phenolic content by HPLC (TPC_HPLC_) and agrimoniin content (AGN)

| Variation Source | DG | PAL | PPO | TPC | TPC_HPLC_ | AGN |
| --- | --- | --- | --- | --- | --- | --- |
| UVA | 1 | *** | ** | ns | *** | *** |
| ST | 1 | ** | * | ns | *** | ns |
| TM | 1 | *** | *** | *** | ** | *** |
| UVA x ST | 1 | ns | ns | ns | ns | ns |
| UVA x TM | 1 | ns | *** | ns | ns | ** |
| ST x TM | 1 | ns | ns | ns | ** | ** |
| UVA ^2^ | 1 | * | ns | ns | ns | * |
| ST ^2^ | 1 | ns | ** | ns | ns | ns |
| TM ^2^ | 1 | ** | ns | *** | *** | *** |
| UVA x ST x TM | 1 | ns | ns | ns | ns | ns |
| UVA^2^ x ST | 1 | ns | ns | ns | ns | ns |
| UVA^2^ x TM | 1 | ns | ns | ns | ns | ns |
| UVA x ST ^2^ | 1 | ns | ns | ns | ns | * |
| UVA x TM ^2^ | 1 | ns | * | * | ns | ns |
| ST ^2^ x TM | 1 | * | ns | ns | ns | ns |
| ST x TM^2^ | 1 | ns | ns | ns | ns | ns |
| R^2^ | - | 0.74 | 0.62 | 0.77 | 0.89 | 0.85 |
| Lack of Fit | 38 | ns | ns | ns | ns | ns |

UVA: UVA radiation dose. ST: Storage temperature, TM: Storage time, DG: degree of freedom. *: p ≤ 0.05; **: p ≤ 0.01; ***: p ≤ 0.001. ns: p> 0.05.

**Reduced quadratic model equations from biofortification process**:

Equation S-1

$$PAL=0.013127+0.014635*UVA+2.63253E-003*ST+1.48128E-003*TM-1.03376E-003*UVA*ST-5.61545E-004*UVA*TM-4.97877E-004*ST*TM+6.49115E-003*{UVA}^{2}-1.82129E-005*{ST}^{2}+1.05613E-004*{TM}^{2}+4.00685E-005*UVA*ST*ST+1.15440E-005*{ST}^{2}*TM+2,56038E-006*ST*{TM}^{2}$$

Equation S-2

$$PPO=0.078182-0.064746*UVA+0.029295*ST+2.53073E-003*TM-3.72425E-003*UVA*TM+0.14114*{UVA}^{2}-1.65425E-003*{ST}^{2}-2.64451E-005*{TM}^{2}+4.66215E-004*{UVA}^{2}*TM+2.0455E-005*UVA*{TM}^{2}$$

Equation S-3

$${TPC}_{HPLC}=1.83114+0.16807*UVA-0.013167*ST-0.023120*TM+1.00621E-003*ST*TM+0.040032*{UVA}^{2}+9.4535E-004*{TM}^{2}$$

Equation S-4

$$TPC=5.27341+4.16250*UVA+0.016903*ST+0.17214*TM-0.048111*UVA*ST+0.027279*UVA*TM-2.9420E-003*ST*TM-2.78729*{UVA}^{2}-7.826669E-004*{TM}^{2}+2.75507E-003*UVA*ST*TM-6.1445E-004*UVA*{TM}^{2}$$

Equation S-5

$$AGN=0.96636+0.15784*UVA-0.096122*ST-1.4479E-003*TM+0.045193*UVA*ST-2.39936E-003*UVA*TM-4.02024E-004*ST*TM-0.052114*{UVA}^{2}+6.00997E-003*{ST}^{2}+6.29268E-004*{TM}^{2}+0.010156*{UVA}^{2}*ST-3.91748E-003*UVA*{ST}^{2}+1.36979E-005*{ST*TM}^{2}$$
